# Using Dalbavancin for the Treatment of Acute Bacterial Skin and Skin Structure Infections (ABSSSIs) in Obese Patients: A Real-Life, Single-Center Observational Study

**DOI:** 10.3390/antibiotics14010075

**Published:** 2025-01-12

**Authors:** Alessandra Oliva, Flavia Petrucci, Cristiana Leanza, Marco Rivano Capparuccia, Michela Comi, Claudio Mastroianni

**Affiliations:** 1 Department of Public Health and Infectious Diseases, Sapienza University of Rome, 00161 Rome, Italy; flavia.petrucci@uniroma1.it (F.P.); cristiana.leanza@uniroma1.it (C.L.); michela.comi@uniroma1.it (M.C.); claudio.mastroianni@uniroma1.it (C.M.); 2 UOC Malattie Infettive, AOU Policlinico “Umberto I”, 00161 Rome, Italy; marco.rivanocapparuccia@uniroma1.it

**Keywords:** dalbavancin, ABSSSI, obese patients, obesity

## Abstract

**Background/Objectives**: Obesity is an established risk factor for several infective conditions, including Acute Bacterial Skin and Skin Structure Infections (ABSSSIs), with a rising trend in their incidence expected in this population. Although numerous antibiotics are available for the prevention and treatment of ABSSSIs, their characterization in obese patients is not a regulatory mandate, highlighting a knowledge gap in this field. Dalbavancin (DAL) is the first approved long-acting antibiotic for the treatment of ABSSSIs. The aim of the study was to describe the clinical effectiveness of DAL in the treatment of ABSSSI, with or without concomitant osteoarticular infections (OAIs), in obese patients compared with non-obese patients. Furthermore, we compared the effectiveness of DAL and intravenous standard of care (SOC) regimens in a subgroup of obese patients with ABSSSI. **Results**: Overall, 45 subjects treated with DAL (12 obese and 33 non-obese) and 8 obese subjects treated with SOC regimens (1:1 ratio) were included. Obese patients treated with DAL had a similar clinical resolution to non-obese patients. However, obese patients tended to have a better cure rate in ABSSSI than OAI. The subgroup of obese patients with ABSSSI had a high clinical resolution, which was comparable to that of SOC. DAL was overall highly tolerated in obese patients. **Methods**: Over a three-year period, hospitalized subjects with ABSSSI who were treated with DAL were included. Patients were further divided into two groups according to the presence/absence of obesity (BMI ≥ 30 kg/m^2^). Furthermore, obese patients treated with DAL were compared with obese patients treated with SOC (1:1 ratio). **Conclusions**: In our real-world study, DAL confirmed its high effectiveness in the treatment of ABSSSI, including in a difficult-to-treat population such as obese patients.

## 1. Introduction

Obesity, defined as body mass index (BMI) ≥ 30 kg/m^2^, represents a unique population that is rapidly growing worldwide and accounts for up to 25–30% of the total population. While higher prevalence has been described in the USA (up to 42.4% of the population), in Italy it is estimated that 10–20% of the population is obese [1,2,3]. However, the prevalence of overweight and obese individuals is expected to rise in the future, posing several therapeutic challenges, especially in terms of optimal dose schedules [4,5]. Indeed, obesity is recognized as a global pandemic by the World Health Organization (WHO), with an estimation that about 60% of the world’s population will be classified as overweight or obese by 2030, based on the BMI scale [2].

Obese patients are at higher risk of infective episodes and worse outcomes than non-obese patients, resulting in more prolonged antimicrobial therapies, hospitalizations and increased treatment costs [1,4,6,7]. Furthermore, the difficulty in finding an adequate venous access in obese patients renders intravenous antimicrobial therapy even more challenging.

Acute Bacterial Skin and Skin Structure Infections (ABSSSIs) [8,9], a subset of complicated skin and soft tissue infections, are prevalent in the obese population, and are often associated with worse outcomes and delayed clinical responses in this specific population [10,11]. Compromised skin integrity, a lower immune response to infection and alteration in the peripheral vascular and nervous functions, all conditions which are present in obese patients, predispose to the development of cutaneous infections [12]. The concomitant presence of diabetes in obese patients, which may be observed in up to 12% of obese patients with ABSSSI [13], may potentiate the increased susceptibility of obese patients to infections, and therefore concur to worsen the outcome [4,6].

Furthermore, obesity can affect the pharmacokinetics (PK) profiles of antimicrobials, by potentially increasing a drug’s volume of distribution, lowering the maximum plasma concentration and enhancing clearance, thus accounting for drugs’ possible underexposure [14,15].

Although numerous antibiotics have been made available for the treatment of ABSSSIs, their characterization in obese patients is not a regulatory mandate, and specific clinical trials investigating their efficacy in obese subjects are lacking. Consequently, information that carries important data for optimizing the dosing regimen specifically targeted at the obese population may not be readily available from trials [16]. This is especially true considering that the approved dosages of several intravenous drugs currently used for the treatment of ABSSSIs, such as daptomycin and glycopeptides, are based on actual/ideal body weight.

Dalbavancin (DAL), a long-acting lipoglycopeptide antibiotic, is approved by the US Food and Drug Administration (FDA) and the European Medicines Agency (EMA) [8,17] for the treatment of ABSSSI in adults and children, with a high clinical effectiveness and an excellent safety profile. DAL characteristics, such as a long half-life, powerful bactericidal activity against the majority of Gram-positive bacteria, high penetration in the bone and the possibility to facilitate patients’ discharge from the hospital, has also encouraged its use for infections other than ABSSSIs, including osteoarticular infections (OAIs), infective endocarditis, implant-associated infections and catheter-related bacteremia [18]. Despite the use of DAL for the treatment of infections other than ABSSSI represents an off-label indication, its use in such conditions has been increasingly supported by real-world studies demonstrating its efficacy in this context, and it is now widely accepted in clinical practice [19]. However, while in the general population evidence on its efficacy has been accumulating over time, data with DAL in obese patients is still limited to post hoc analysis or single case reports [13,20].

Therefore, in a time when a tailored and targeted appropriate antimicrobial therapy is highly desirable, there is still a knowledge gap that needs to be filled concerning the treatment of ABSSSI with DAL in obese patients.

Based on these premises, the aims of the study were to (i) describe the clinical effectiveness of DAL in the treatment of ABSSSI, with or without concomitant OAIs, in obese and non-obese patients and (ii) compare DAL with standard of care (SOC) regimens in the subgroup of obese patients with ABSSSI. Secondary endpoints were to evaluate the length of hospitalization and safety of antimicrobial treatments in obese patients.

## 2. Results

### 2.1. General Characteristics of Study Population

Overall, 45 patients treated with DAL for ABSSSI, with or without concomitant OAIs, were enrolled in the study, including 28 (62.2%) males, with a median age 64 (54–73) years. The median Charlson Comorbidity Index was 3 (1–5), with a concomitant diagnosis of diabetes mellitus in 8 patients (17.7%). Twelve subjects (26.7%) were obese, and 33 (73.3%) were non-obese. All the patients had a diagnosis of ABSSSI, which was associated with a concomitant OAIs in 28 patients (62.2%) [osteomyelitis in 10 (22.2%), spondylodiscitis in 15 (33.3%) and prosthetic joint infection in 3 (6.7%)]. In detail, among the 10 patients with osteomyelitis, 4 involved the tibia, 2 the hand phalanges, 2 the humerus, 1 the astragalus and 1 the fifth metatarsus; among the 15 spondylodiscitis, 12 were lumbar and 3 were dorsal; the 3 prosthetic joint infections involved the hip (n = 1) and the knee (n = 2).

**Figure 1:**
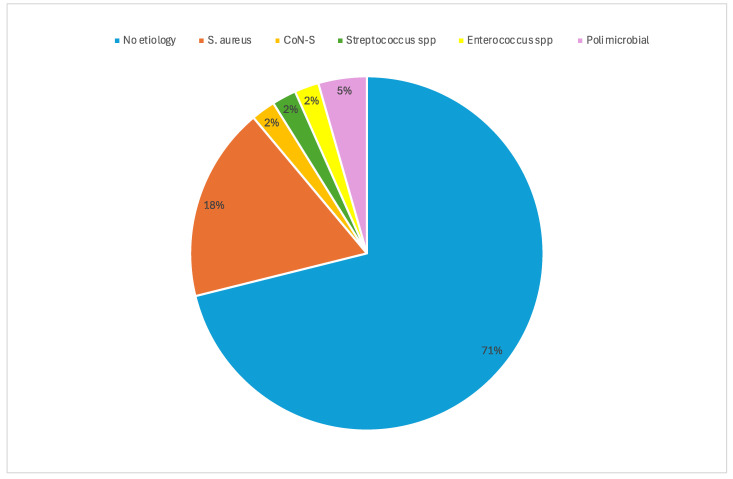
Microbiological characteristics of acute bacterial skin and skin structure infections. CoN-S: coagulase-negative *Staphylococci*. The causative microorganisms were isolated in only a minority of patients (13/45, 28.9%): six methicillin-resistant *Staphylococcus aureus* (MRSA), two methicillin-susceptible *S. aureus* (MSSA), one methicillin-resistant *S. epidermidis* (MRSE), one *Streptococcus* spp. one *Enterococcus faecalis* and two polymicrobial (MSSA + *Pseudomonas aeruginosa*, MSSA + *Proteus mirabilis*).

The causative microorganisms were isolated in only a minority of patients (13/45, 28.9%): six methicillin-resistant *Staphylococcus aureus* (MRSA), two methicillin-susceptible *S. aureus* (MSSA), one methicillin-resistant *S. epidermidis* (MRSE), one *Streptococcus* spp., one *Enterococcus faecalis* and two polymicrobial (MSSA + *Pseudomonas aeruginosa*, MSSA + *Proteus mirabilis*) (Figure 1).

The mean number of DAL infusions was 1.82 (±0.98). DAL was used as the first therapeutic scheme in two patients (4.4%), while in the majority of cases, DAL was the second or the third therapeutic choice (30, 66.7% and 13, 28.9%, respectively). The previous antibiotic therapies were daptomycin (26 patients, 59%), glycopeptides (15 patients, 34%), linezolid (3 patients, 6.8%), fluoroquinolones (15, 34%) or beta-lactams/beta-lactamases inhibitors (15, 34%).

DAL was administered as follows: 1500 mg single dose (31 patients, 68.8%), 1000 mg day 1 followed by 500 mg day 8 (7 patients, 15.5%), 1500 mg followed by 1000 mg and 500 mg (6 patients, 13.4%), 1000 mg single dose (1 patient, 2.3%) (Table 1). Only three patients did not receive DAL as monotherapy, instead being treated in combination with fluoroquinolones for the entire duration of the DAL therapy.

As expected, DAL was used as a therapeutic switch to favor hospital discharge in 42/45 (93.3%) patients, while it was only used in three patients (6.7%) after the therapeutic failure of other regimens.

Clinical cure/improvement rates at 7 and 14 days were 51.1% (23/45) and 91.1% (41/45), which were maintained even when concomitant OAI was present (46.4% and 89.3%, respectively), while at the 30-day follow-up, the clinical cure/improvement was 91.1% (41/45), 89.3% (25/28) if concomitant OAIs were present. The follow-up at 8 weeks in patients with concomitant OAIs showed a clinical cure in 24/28 (85.7%).

Infection relapse was observed in four patients (8.9%). Overall, DAL was well tolerated, with adverse effects of mild entity only observed in one obese patient (2.2%) (nausea).

### 2.2. Comparison Between Obese and Non-Obese Patients

A comparison between obese and non-obese patients treated with DAL is shown in Table 1.

**Figure 2:**
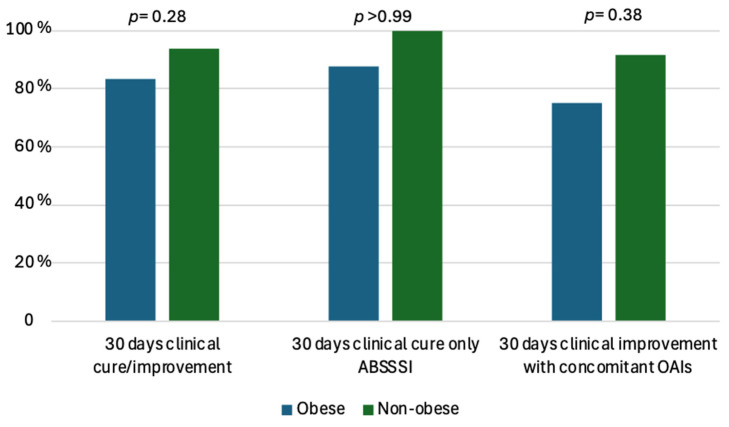
Overall clinical cure/improvement at 30 days and stratified by diagnosis (ABSSI alone or concomitant OAIs) in obese and non-obese patients. ABSSSI: acute bacterial skin and skin structure infections; OAI: osteoarticular infections.

The median BMI in obese patients was 35 (31.5–36.7), while in non-obese patients, it was 24 (22.5–25.5) (*p* < 0.0001). ABSSSI alone (without concomitant OAIs, n = 17) was more common in obese than non-obese patients (8/12, 66.7% vs. 9/33, 27.3%, *p* = 0.03). Diabetes was more common in obese than non-obese patients (5/12, 41.7% vs. 3/33, 9.1%, *p* = 0.02). Median inflammatory values (CRP, WBC) and fever were similar between the two groups. A previous antibiotic therapy was administered for 20 (11.7–28) and 20 (12–28) days in obese and non-obese patients, respectively (*p* = 0.97). Likewise, no difference was observed for the mean number of DAL administrations (1.41 vs. 1.97 in obese and non-obese patients, respectively, *p* = 0.16). The primary reason for DAL use was to favor hospital discharge, with a slightly lower proportion in obese patients (10/12, 83.3%) compared to non-obese patients (32/33, 96.9%), while DAL use due to failure of other regimens was more common in obese patients (1/12, 16.6% vs. 2/33, 3.03%), though these differences are not statistically significant (*p* = 0.16). No differences were observed between the two groups for the modality of DAL administration [1 (8.3%), 7 (58.3%), 5 (41.6%) vs. 1 (3.1%), 23 (68.7%), 8 (24.2%) as the first, second or third therapeutic choice, respectively (*p* = 0.46, *p* = 0.72, *p* = 0.28, respectively)]. A similar length of hospitalization was observed in the two groups (22.5 ± 16.7 vs. 24.8 ± 14.1 days in obese and non-obese subjects, respectively). Clinical cure/improvement at 7 and 14 days was similar between the two groups: 5/12 (41.6%) vs. 18/33 (54.5%) (*p* = 0.51) and 11/12 (91.6%) vs. 30/33 (90.9%) (*p* > 0.99) in obese and non-obese patients, respectively. Clinical improvement in cases with OAIs at 7 and 14 days was similar between the two groups: 1/4 (25%) vs. 12/24 (50%) (*p* = 0.60) and 4/4 (100%) vs. 21/24 (87.5%) (*p* > 0.99) in obese and non-obese patients, respectively. At 30-day follow-up, clinical cure/improvement was slightly higher in non-obese than obese patients [31/33 (93.9%) vs. 10/12 (83.3%), *p* = 0.28]. After stratifying for diagnosis, clinical resolution at 30 d was 7/8 (87.5%) and 9/9 (100%) in obese and non-obese patients with ABSSSIs alone, respectively (*p* >0.99); when a concomitant OAI was present, clinical improvement was slightly lower in obese (3/4, 75%) than in non-obese patients (22/24, 91.7%), although this was not statistically significant (*p* = 0.38) (Figure 2). Clinical relapse was higher in obese than non-obese patients (2, 16.7% vs. 2, 6%, *p* = 0.28).

The obese patients with and without diabetes had similar clinical outcomes (40% vs. 42.8% at 7 days, 100% vs. 85.7% at 14 days, 20% vs. 28.57% at 30 days, respectively) (Table 2).

### 2.3. Comparison Between DAL and SOC in Obese Patients with ABSSSI Alone

The subset of obese patients treated with DAL for ABSSSI alone (n = 8) was compared with eight obese patients with ABSSSI who were only treated with SOC (Table 3).

As expected, no difference in BMI was found between the two groups (36.7 ± 4.8 vs. 36.9 ± 2.7 in DAL and SOC, respectively, *p* = 0.94). The mean age of the DAL subjects was 68.5 ± 9.6 years, higher than SOC (50.7 ± 12.1 years, *p* = 0.005). Diabetes was present in four patients (50%) with SOC and in three patients (37.5%) with DAL. The length of hospitalization was similar in the groups (22.5 ± 20 vs. 21 ± 7.9 days, *p* = 0.84).

**Figure 3:**
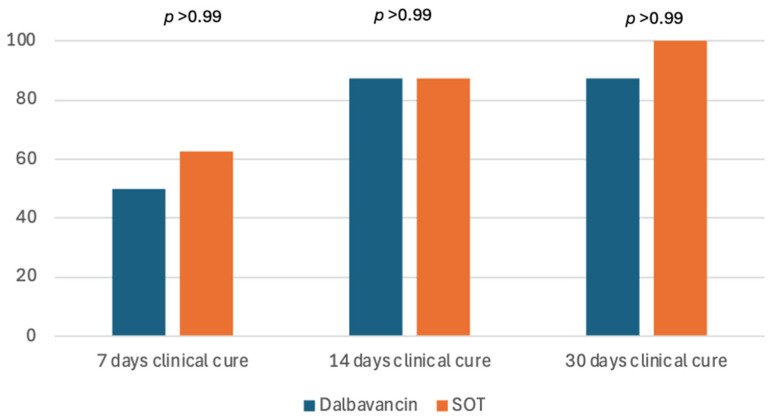
Clinical cure at 7, 14 and 30 days stratified by therapy (Dalbavancin or SOC) in obese patients with acute bacterial skin and skin structure infections. SOC: standard of care.

Clinical cure at seven days was higher, albeit not statistically significant, in obese patients treated with SOC than DAL (5/8, 62.5% vs. 4/8, 50% *p* > 0.99), while at 14- and 30-day follow up, the resolution was similar in both groups (87.5% for both the DAL and SOC groups at 14 days, 87.5% and 100% for the DAL and SOC groups at 30 days, *p* > 0.99 each) (Figure 3).

DAL was administered as the first choice for one subject, as second for four patients and as third for three subjects. The used DAL dosages were: 1500 mg single dose (six patients), 1000 mg day 1 and 500 mg day 8 (one patient), single dose 1000 mg (one patient). SOC regimens consisted of daptomycin (six patients, dosage ranging from 500 to 1000 mg daily), linezolid (one patient), tigecycline (one patient). Adverse events in the SOC group were observed in two subjects and were of mild entity.

## 3. Discussion

In this real-life study, we investigated the clinical effectiveness of DAL in obese patients for the treatment of ABSSSI, with or without concomitant OAIs. Furthermore, to evaluate potential differences between DAL and other therapeutic schemes in the subgroup of obese patients with ABSSSI only, we compared obese patients treated with DAL or SOC.

Overall, we found that (i) obese patients treated with DAL had a similar clinical resolution to non-obese patients; (ii) obese patients treated with DAL tended to be better cured in ABSSSI than OAI groups; (iii) the subgroup of obese patients with ABSSSI had a high clinical resolution, similar to that of SOC and (iv) DAL was highly tolerated also in obese patients.

To the best of our knowledge, this is the largest real-life study on DAL treatment in obese patients. As a matter of fact, so far, the majority of data in this population comes from a recent post hoc analysis of phase III trials comparing DAL with vancomycin or DAL one dose versus DAL two doses in ABSSSI: authors found similar clinical success rates at 48–72 h and at end of treatment in patients with normal or elevated BMI (89.3% and 90.9% versus 87.6% and 95.2%, respectively), with a similar safety profile across patient groups, suggesting that treatment of ABSSSI with DAL is effective even in patients with high BMI [13].

Only a few real-life studies have described the BMI of the study population. In the study by Bai et al., the median BMI was 24.97, with a BMI higher than 30 only observed in the ABSSSI group; however, data regarding outcomes in patients with obesity were not present [21]. Ramadan et al. described a case series of 13 patients with spondylodiscitis who were treated with DAL, with a mean BMI of 31 (SD 6) and a clinical success achieved in 11/13 (84.6%); however, no detailed description in patients with BMI > 30 could be found [22]. More recently, a single case report showed clinical failure under DAL treatment for MRSA bacteremia in a patient with severe obesity (BMI 66) and intravenous drug abuse [20].

Another strength of our report, although limited by the small number of patients, is represented by the direct 1:1 comparison of obese patients with ABSSSI who were only treated with DAL or SOC; even in this case, we found similar high clinical cure rates between the two groups.

Obesity, which has been recognized as a global pandemic by the WHO, with estimation of up to 60% of prevalence by 2030 [2], is a well-recognized risk factor for infections and is often associated with a slower clinical resolution than non-obese patients. Furthermore, obesity was associated with treatment failure in hospitalized patients with cellulitis [3].

As for infection management, obese patients are more likely to receive antimicrobial therapy regimens in a hospital setting, with important clinical and economic consequences, particularly as obesity is increasing in prevalence. Furthermore, the prolonged hospitalization associated with obesity may favor the risk of nosocomial infection and antimicrobial resistance acquisition. Lastly, the paucity of venous accesses, a common condition in obesity, renders the antibiotic therapy even more challenging.

The causes of increased infection risk in obesity are various and diverse. Despite increased susceptibility for co-morbidities such as type 2 diabetes, obesity per se is associated with altered cytokine synthesis, reduced antigen response and the diminished function of natural killer cells, dendritic cells and macrophages. The disruption of lymphoid tissue integrity by fat accumulation and altered secretion of adipocytokines, such as leptin or adiponectin, has been suggested to explain immune dysfunction and infection susceptibility in obese patients [15]. Adipose tissue can act as an endocrine organ and exert immunomodulatory effects on the body. Although the full effect of this immunomodulation in obesity is unknown, it is plausible that it may be a contributing factor to the observed worse outcomes of infection among obese people [23].

One additional factor contributing to the observed poorer infection outcomes among obese patients may be inadequate antimicrobial dosing due the lack of weight-related dosing adjustment, and the paucity of evidence on dosing due to lack of antimicrobial pharmacokinetic studies. Indeed, obesity can negatively affect the PK profiles of antimicrobials by potentially increasing a drug’s volume of distribution, lowering the maximum plasma concentration and enhancing drug clearance [14]. Furthermore, the antimicrobial penetration rate into the subcutaneous tissue involved during skin infection plays a pivotal role in determining the outcome. In this regard, data concerning the PK/PD of DAL in the obese population is very scarce. Buckwalter et al. performed a population PK study and showed a significant linear relationship between DAL clearance and body surface area (BSA) and creatinine clearance, while the drug’s volume of distribution was influenced by BSA alone [24]. The final model indicated that patients with higher BSA had an approximately one third decrease in DAL Cmax [24]. However, a more recent study showed only a minor reduction in Cmax and AUC as the BSA and patients’ weight increased. Based on the simulation, authors concluded that dose adjustment of DAL in obesity is unlikely to be necessary, though there is limited clinical experience with DAL in this population [14]. Our results are in line with the latter conclusion, since non-weight adjusted dosages of DAL in obese patients achieved similar outcomes of non-obese subjects. Although we could not verify this assumption since we did not perform DAL serum concentration in our patients, our encouraging findings, along with the results of the post hoc analysis, seem to confirm the favorable profile of DAL in the obese population, and could open the path to additional investigations on DAL’s optimal use in this unique population. Indeed, DAL’s features (long half-life which permits only few drug administration and may avoid the need of central venous accesses positioning, low level of drug–drug interaction, high activity against Gram-positive bacteria and excellent safety profile) contribute to rendering DAL an optimal therapeutic choice, even in obese patients [18].

Undoubtedly, the present study has several limitations that should be underlined. First, the retrospective and single-center nature of the study, as well as the low number of enrolled patients, could not lead to a generalization of the results; second, the definite etiology of infection was possible only in a minority of subjects, and therefore we could not evaluate whether DAL effectiveness differed according to the pathogens; third, we did not perform the therapeutic dosage of DAL, and therefore we could not assess whether the clinical observed responses depended on PK/PD parameters; fourth, the choice of DAL therapy, including its dosing, scheme, use in combination and duration and use for primary or follow-on therapy, was not uniform in all the patients, but rather it was based on the clinical judgment of treating physicians. DAL MIC was not performed and complete data regarding surgical interventions for patients with osteomyelitis and prosthetic joint infection were not available. Moreover, since only one patient had a BMI > 40, our conclusions should be restricted to obese patients with BMI < 40. However, we believe that additional studies evaluating the effectiveness of DAL, specifically targeting obese patients in a real-life setting will give additional insights into the potential use of this drug for this high-risk population. Indeed, reducing the length of hospital stay, as well as the potential complications of prolonged intravenous antibiotics, may represent additional advantages of DAL administration.

## 4. Materials and Methods

### 4.1. Study Design

This was a non-interventional, retrospective, single-center study, including all consecutive hospitalized adult patients with ABSSSIs, with or without concomitant OAIs, requiring intravenous treatment and treated with DAL, either as a first choice or as a switch strategy after previous antimicrobial treatment.

Inclusion criteria included (i) age ≥ 18 years, (ii) diagnosis of ABSSSI, with or without concomitant OAIs and (iii) the need of intravenous antimicrobials for ABSSSI treatment. Exclusion criteria were age < 18 years, skin and soft tissue infections other than ABSSSI and lack of data.

**Figure 4:**
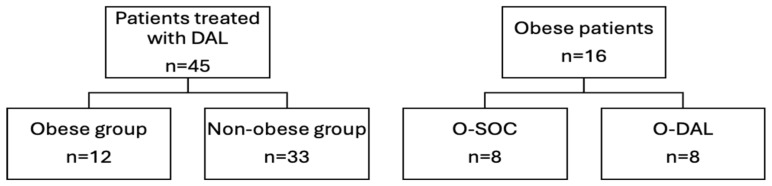
Study population DAL: dalbavancin; SOC: standard of care; O-SOC: obese patients treated with standard of care; O-DAL: obese patients treated with Dalbavancin.

The study population was further divided according to the presence of obesity (obese versus non-obese group) and, amongst obese patients, according to the received treatment, namely DAL or SOC (O-DAL and O-SOC groups, respectively, 1:1 ratio) (Figure 4).

The study was approved by the local Ethical Committee (58/2021). Patients gave consent for data sharing for research purposes upon hospital admission. Being a retrospective study, specific informed consent was not required.

### 4.2. Definitions

ABSSSI were defined according to guidelines and the FDA definition [8]. Microbiological samples were obtained from deep tissue and, in case of OAI, from bone and/or tissue biopsies.

Clinical cure was defined as local plus systemic improvement at 7 and 14 days after the institution of intravenous antimicrobial treatment with DAL. Local improvement was defined as a reduction in inflammatory signs/symptoms (i.e., diameter of skin involvement, redness, swelling, wound dehiscence, pain) and/or purulent secretion based on clinical evaluation/judgment for clinical practice, whereas systemic improvement included reduction in fever and/or sepsis signs (when present), as well as a reduction in inflammatory markers [C-reactive protein (CRP), white blood cells (WBCs), procalcitonin (PCT), when performed]. Sepsis was defined according to SEPSIS-3 definitions [25]. Obesity was defined as a body mass index (BMI) ≥ 30 [12].

Clinical improvement (defined as above) was considered for ABSSSI with concomitant OAIs for 7-, 14- and 30-day follow-up. Relapse was clinically defined as a clinical worsening after an initial clinical improvement.

### 4.3. Antimicrobial Regimens

Given the observational nature of the study, the choice of antimicrobial therapy was based on the clinical judgment of treating physicians.

The decision of starting DAL (first choice or second/third choice after intravenous antibiotic treatment), as well as DAL therapeutic schemes, were collected.

SOC included the intravenous antimicrobial regimens used by physicians for the treatment of ABSSSI in obese patients: daptomycin (6–8 mg/kg every 24 h, or according to renal function), teicoplanin (6–12 mg/kg every 12 h for three doses as loading dose followed by 6–12 mg/kg every 24 h, or according to renal function), vancomycin (20 mg/kg using actual body as loading dose followed by 15 mg/kg every 12 h, with daily dosage not exceeding 3 g), linezolid (600 mg every 12 h), tigecycline (100 mg as loading dose, followed by 50 mg every 12 h).

### 4.4. Data Collection

Demographic (age, Charlson Comorbidity Index, gender, BMI, presence of diabetes mellitus), infection (fever, ABSSSI, presence/absence of concomitant OAIs), laboratory (C-reactive protein; CRP, white blood cell counts, WBC; erythrocyte sedimentation rate, ESR), microbiological (positivity/negativity of blood cultures and/or other collected cultures; type of isolated microorganism; mono- vs. polymicrobial infection) and therapeutic data [number of antimicrobial regimens (first, second, third line of regimen), number of DAL doses, monotherapy or combination therapy, duration of therapy before DAL, reasons for DAL use (therapeutic switch, failure/toxicity of previous therapy), total duration of antimicrobial therapy, adverse events], were collected from clinical charts and anonymously inserted in an electronic database.

### 4.5. Statistical Analyses

Categorical variables were described through absolute frequencies and percentages; quantitative variables were reported through median with interquartile range or mean and SD, depending on the normal or non-normal distribution of the data. Differences between qualitative variables were analyzed by means of Chi-square or Fischer tests, while differences between quantitative variables were assessed by means of t-Student or Mann–Whitney tests, as appropriate. *p*-value analyses were two-sided, and a *p*-value of less than 0.05 was considered statistically significant. All statistical analyses were performed using STATATM software, v. 18 (StataCorp) and Graphpad PrismTM charts, version 10.0.3 (2017) using Microsoft OfficeTM (version 16.77.1) and Graphpad PrismTM.

## 5. Conclusions

DAL was highly effective and safe in obese patients with ABSSSI, with or without concomitant OAIs, suggesting that no weight-based dosage adjustment would be required in obese patients. Future prospective, multicenter studies specifically targeting this high-risk and difficult-to-treat population are warranted.

## Figures and Tables

**Table 1:** General features of study population. ABSSSI: acute bacterial skin and skin structure infections; BMI: body mass index; CCI: Charlson comorbidity index; CRP: C-reactive protein; DAL: dalbavancin; ESR: erythrocyte sedimentation rate; NA: not applicable; OAI: osteoarticular infections; WBC: white blood cells.

	Total n = 45	Obese n = 12	Non-Obese n = 33	*p*-Value
**Demographics**				
Age, years, median (IQR)	64 (54–73)	66.5 (50.7–75.2)	64 (54–71)	0.91
Gender (male), n (%)	28 (62.2)	7 (58.3)	21 (63.6)	0.4526
BMI, median (IQR)	24 (23–30.5)	35 (31.5–36.75)	24 (22.5–25.5)	<0.0001
Diabetes mellitus, n (%)	8 (17.7)	5 (41.7)	3 (9.1)	0.02
CCI, median (IQR)	3(1–5)	4 (1.2–5)	3 (1–5)	0.51
**Clinical features**				
ABSSSI without bone involvement, n (%) ABSSSI with bone involvement, n (%)	17 (37) 28 (62.2)	8 (66.7) 4 (33.3)	9 (27.3) 24 (72.7)	0.03
Microbiological isolation, n (%)	13 (28.9)	4 (33.3)	9 (27.3)	0.72
Fever °, n (%)	22 (48.9)	7 (58.3)	15 (45.4)	0.51
**Laboratory findings**				
CRP mg/dl at infection onset, median (IQR)	5.23 (0.8–17.2)	2.5 (0.6–30.3)	5.41 (0.8–14.1)	0.82
ESR at infection onset, median (IQR)	60 (35–98)	61.5 (24–114)	60 (35–88)	0.84
WBC at infection onset, median (IQR)	9940 (7300–14,105)	8405 (5600–16,008)	10170 (7820–14,030)	0.52
CRP mg/dL before DAL, median (IQR)	1.25 (0.37–5.15)	1.95 (0.37–5.7)	1.2 (0.32–5)	0.87
ESR before DAL, median (IQR)	43 (27–85)	38 (20–51.5)	57 (28–85)	0.30
WBC before DAL, median (IQR)	6395 (5323–8260)	6600 (5245–8025)	6190 (5305–8320)	0.87
**Antibiotic therapy prior to DAL**				
Overall previous therapy duration, days median (IQR)	20 (11.7–28)	20 (11–28)	20 (12–28)	0.97
Daptomycin, n (%)	26 (57.7)	6 (50)	20 (60.6)	0.734
Glycopeptides, n (%)	15 (33.3)	3 (25)	12 (36.4)	0.72
Linezolid, n (%)	3 (6.7)	0 (0)	3 (9.1)	0.55
Fluoroquinolones, n (%)	15 (33.3)	3 (25%)	12 (36.4)	0.72
β-lactam/β-lactamase inhibitors, n (%)	15 (33.3)	6 (50%)	9 (27.3)	0.17
**DAL therapy**				
Number of DAL administration, mean (± SD)	1.82 ± 0.98	1.41 ± 0.51	1.97 ± 1.07	0.16
Reason for DAL use: favor hospital discharge, n (%)	42 (93.3)	10 (83.3)	32 (96.9)	0.16
Reason for DAL use: failure of other regimens, n (%)	3 (6.7)	2 (16.6)	1 (3.03)	0.16
Dalbavancin as 1st option, n (%)	2 (4.4)	1 (8.3)	1 (3.1)	0.46
Dalbavancin as 2nd option, n (%)	30 (66.7)	7 (58.3)	23 (69.7)	0.72
Dalbavancin as 3rd option, n (%)	13 (28.9)	5 (41.6)	8 (24.2)	0.28
DAL 1500 mg single dose, n (%)	31 (69.8)	10 (83.3)	21 (63.6)	0.45
DAL 1000 mg + 500 mg, n (%)	7 (15.5)	2 (16.7)	5 (15.1)	>0.99
DAL 1500 mg + 1000 mg + 500 mg, n (%)	6 (13.3)	0 (0)	6 (18.2)	0.16
DAL 1000 mg, n (%)	1 (2.2)	0 (0)	1 (3.0)	0.16
**Outcomes**				
7 days clinical cure */improvement **, n (%) 7 days clinical cure, ABSSSI alone (total n = 17), n (%) 7 days clinical improvement ** with concomitant OAIs (total n = 28), n (%)	23 (51.1) 10/17 (58.8) 13/28 (46.4)	5 (41.6) 4/8 (50) 1/4 (25)	18 (54.5) 6/9 (66.6) 12/24 (50)	0.51 0.63 0.60
14 days clinical cure */improvement **, n (%) 14 days clinical cure, ABSSSI alone (total n = 17), n (%) 14 days clinical improvement ** with concomitant OAIs (total n = 28), n (%)	41 (91.1) 16/17 (94.1) 25/28 (89.3)	11 (91.6) 7/8 (87.5) 4/4 (100)	30 (90.9) 9/9 (100) 21/24 (87.5)	>0.99 0.47 >0.99
30 days clinical cure */improvement **, n (%) 30 days clinical cure *, ABSSSI alone (total n = 17), n (%) 30 days clinical improvement ** with concomitant OAIs (total n = 28), n (%)	41 (91.1) 16/17 (94.1) 25/28 (89.3)	10 (83.3) 7/8 (87.5) 3/4 (75)	31 (93.9) 9/9 (100) 22/24 (91.7)	0.28 >0.99 0.38
8 weeks clinical cure * with concomitant OAIs (total n = 28), n (%)	24/28 (85.7)	3/4 (75)	21/24 (87.5)	0.48
30 days outcome: relapse ***, n (%)	4 (8.9)	2 (16.7)	2 (6)	0.28
Length of hospitalization (days, median IQR)	21.7 ± 14.7	22.5 ± 16.7	24.8 ± 14.1	0.84
Adverse effects, n (%)	1 (2.2)	1 (8.3)	0	NA

°: at hospital admission. *: Clinical cure was defined as local plus systemic improvement at 7 and 14 days after the institution of intravenous antimicrobial treatment with DAL. Local improvement was defined as a reduction in inflammatory signs/symptoms (i.e., diameter of skin involvement, redness, swelling, wound dehiscence, pain) and/or purulent secretion based on clinical evaluation/judgment as for clinical practice, whereas systemic improvement included reduction in fever and/or sepsis signs (when present), as well as a reduction in inflammatory markers [C-reactive protein (CRP), normal values: 0–0.5 mg/dL; white blood cells (WBCs), normal values 4.00–10.00 × 10^3^/µL and erythrocyte sedimentation rate (ESR), normal values 0–25 mm/h, when performed]. **: Clinical improvement (defined as above) was considered for ABSSSI with concomitant OAIs for 7-, 14- and 30-day follow-up. ***: Relapse was clinically defined as a clinical worsening after an initial clinical improvement.

**Table 2:** Outcomes between obese and non-obese population with diabetes.

	Total n = 12	Obese with Diabetes n = 5	Non-Obese with Diabetes n = 7	*p*-Value
7 days clinical cure */improvement **, n (%)	5 (41.6)	2 (40)	3 (42.8)	>0.999
14 days clinical cure */improvement **, n (%)	11 (91.6)	5 (100)	6 (85.7)	>0.999
30 days clinical cure */improvement **, n (%)	3 (25)	1 (20)	2 (28.6)	>0.999
30 days outcome: relapse ***, n (%)	2 (16.7)	0 (0)	2 (28.6)	0.469

*: Clinical cure was defined as local plus systemic improvement at 7 and 14 days after the institution of intravenous antimicrobial treatment with DAL. Local improvement was defined as a reduction in inflammatory signs/symptoms (i.e., diameter of skin involvement, redness, swelling, wound dehiscence, pain) and/or purulent secretion based on clinical evaluation/judgment as for clinical practice, whereas systemic improvement included reduction in fever and/or sepsis signs (when present), as well as a reduction in inflammatory markers [C-reactive protein (CRP), white blood cells (WBCs), procalcitonin (PCT), when performed]. **: clinical improvement (defined as above) was considered for ABSSSI with concomitant OAIs for 7-, 14- and 30-day follow-up. ***: relapse was clinically defined as a clinical worsening after an initial clinical improvement.

**Table 3:** Comparison of obese patients treated with DAL or with SOC. BMI: body mass index.

	Total n = 16	Dalbavancin ^§^ n = 8	SOC ° n = 8	*p*-Value
Age, years median (IQR)	57.6 ± 15.9	68.5 ± 9.6	50.7 ± 12	0.005
BMI, median (IQR)	36.7 ± 3.6	36.7 ± 4.8	36.9 ± 2.7	0.94
Diabetes mellitus, n (%)	7 (30)	3 (37.5)	4 (50)	>0.99
7 days clinical cure *, n (%)	9 (56.2)	4 (50)	5 (62.5)	>0.99
14 days clinical cure *, n (%)	14 (87.5)	7 (87.5)	7 (87.5)	>0.99
30 days clinical cure *, n (%)	15 (93.7)	7 (87.5)	8 (100)	>0.99
Length of hospitalization, days (±standard deviation)	21.7 ± 12.4	22.5 ± 20	21 ± 7.9	0.845

*: Clinical cure was defined as local plus systemic improvement at 7 and 14 days after the institution of intravenous antimicrobial treatment with DAL. Local improvement was defined as a reduction in inflammatory signs/symptoms (i.e., diameter of skin involvement, redness, swelling, wound dehiscence, pain) and/or purulent secretion based on clinical evaluation/judgment as for clinical practice, whereas systemic improvement included reduction in fever and/or sepsis signs (when present), as well as a reduction in inflammatory markers [C-reactive protein (CRP), white blood cells (WBCs), procalcitonin (PCT), when performed]. ^§^: The used DAL dosages were: 1500 mg single dose (six patients), 1000 mg day 1 and 500 mg day 8 (one patient), single dose 1000 mg (one patient). °: SOC regimens consisted of daptomycin (six patients, dosage ranging from 500 to 1000 mg daily), linezolid (one patient), tigecycline (one patient). Adverse events in the SOC group were observed in two subjects and were of mild entity.

## Data Availability

Data are unavailable due to privacy restrictions.
